# L-fucose and fucoidan alleviate high-salt diet-promoted acute inflammation

**DOI:** 10.3389/fimmu.2024.1333848

**Published:** 2024-03-26

**Authors:** Wenhua Li, Pengfei Wu, Tianrong Jin, Jialin Jia, Bo Chen, Tingting Liu, Yu Liu, Jie Mei, Bangwei Luo, Zhiren Zhang

**Affiliations:** ^1^ Institute of Immunology, Third Military Medical University, Chongqing, China; ^2^ Research Center of Integrative Medicine, School of Basic Medical Sciences, Guangzhou University of Chinese Medicine, Guangzhou, China; ^3^ Department of Pulmonary and Critical Care Medicine, Institute of Respiratory Diseases, Xinqiao Hospital, Third Military Medical University, Chongqing, China; ^4^ Medical College of Chongqing University, Chongqing, China; ^5^ College of Acupuncture and Tuina, Guizhou University of Traditional Chinese Medicine, Guiyang, Guizhou, China

**Keywords:** macrophage, inflammation resolution, microbiota, L-fucose, fucoidan, high salt diet

## Abstract

Excessive salt intake is a widespread health issue observed in almost every country around the world. A high salt diet (HSD) has a strong correlation with numerous diseases, including hypertension, chronic kidney disease, and autoimmune disorders. However, the mechanisms underlying HSD-promotion of inflammation and exacerbation of these diseases are not fully understood. In this study, we observed that HSD consumption reduced the abundance of the gut microbial metabolite L-fucose, leading to a more substantial inflammatory response in mice. A HSD led to increased peritonitis incidence in mice, as evidenced by the increased accumulation of inflammatory cells and elevated levels of inflammatory cytokines, such as tumor necrosis factor alpha (TNF-α), interleukin 6 (IL-6), and monocyte chemotactic protein-1 (MCP-1, also known as C-C motif chemokine ligand 2 or CCL2), in peritoneal lavage fluid. Following the administration of broad-spectrum antibiotics, HSD-induced inflammation was abolished, indicating that the proinflammatory effects of HSD were not due to the direct effect of sodium, but rather to HSD-induced alterations in the composition of the gut microbiota. By using untargeted metabolomics techniques, we determined that the levels of the gut microbial metabolite L-fucose were reduced by a HSD. Moreover, the administration of L-fucose or fucoidan, a compound derived from brown that is rich in L-fucose, normalized the level of inflammation in mice following HSD induction. In addition, both L-fucose and fucoidan inhibited LPS-induced macrophage activation *in vitro*. In summary, our research showed that reduced L-fucose levels in the gut contributed to HSD-exacerbated acute inflammation in mice; these results indicate that L-fucose and fucoidan could interfere with HSD-promotion of the inflammatory response.

## Introduction

Salt is not only a basic condiment but also a substance that is essential for physical development. The main component of salt is sodium chloride (NaCl), which plays a crucial role in various biological processes, such as osmotic pressure, on the generation of hydrogen potential, and electroneurophysiology (1–8). However, with the improvements in living standards, salt intake has significantly increased. John Powles showed that sodium intake in 181 out of the 187 countries, whose total adult population accounted for 99.2% of the world’s adult population, exceeded the World Health Organization (WHO) recommendation of 2.00 g/day sodium (equivalent to 5 g/day of salt) in 2010 (9). The excessive consumption of salt is strongly associated with the development and progression of many diseases. A study conducted across 195 countries from 1990 to 2017 revealed that high sodium intake was the leading dietary risk factor for death and disability-adjusted life-years (DALYs). In 2017 alone, high sodium intake was responsible for 3 million deaths and 70 million DALYs (10).

A pro-inflammatory effect is one of the significant mechanisms through which a HSD promotes the occurrence and development of HSD-related diseases. However, how salt alters the inflammatory response *in vivo* is not fully understood. Accumulated data have shown that sodium chloride, the primary component of salt, can accumulate in some parts of the body and directly amplify inflammatory activation and the lymphocyte response, leading to an inflammatory state in the body (11–17). Moreover, recent findings have shown no significant differences in sodium chloride levels in the serum, heart or lungs of HSD-fed and chow diet (CD)-fed mice (18) and a HSD can affect systemic inflammatory responses by altering the gut microbiota and its metabolites in mice and humans (11).

The intestinal microbiota begins to shape and grow at birth and gradually matures in response to exposure to various environmental factors. The gut microbiota consists of more than 50 different phyla that contain more than 1500 species (19). A healthy gut microbiota is characterized by high taxonomic diversity, high levels of microbial gene richness and stable functional cores of the microbiome (19, 20). Recent research has suggested that the intestinal microbiota and its metabolic byproducts have a significant impact on the health of the host. A deviation in the composition or function of the microbiota from that of the healthy state can lead to metabolic disorders (20, 21). Moreover, the intestinal microbiota can be influenced by various factors, including lifestyle, medication, diet, genetics, and so on (19, 20). Extensive studies on the gut microbiota have demonstrated that diet may alter the microbial composition and function in humans and other mammals (22). A recent study showed that HSD consumption increased the ratio of *Firmicutes/Bacteroidetes*, and the abundances of the genera *Lachnospiraceae* and *Ruminococcus* while reducing the abundance of *Lactobacillus* (23). Another study demonstrated that HSD consumption reduced the relative abundance of *Lactobacillus* sp., significantly decreased butyrate levels and ultimately worsened dextran sodium sulfate (DSS)-induced colitis in mice fed a HSD compared with those fed a CD (24). Wlick et al. reported that a HSD depletes *Lactobacillus murinus* in the gut and ameliorates salt-induced aggravation of actively induced experimental autoimmune encephalomyelitis and salt-sensitive hypertension by oral administration of *Lactobacillus murinus* (25). Nevertheless, recent studies have focused on the composition of the intestinal microbiota and have focused on *Lactobacillus*-related metabolites, such as short-chain fatty acids and indole metabolites. Therefore, in the present study, we examined the alteration in the composition of the intestinal microbiota after HSD treatment and investigated whether other metabolites play important roles and the relationships among the microbiota. In addition, we investigated the metabolites to investigate their effects on HSD-induced inflammation.

## Results

### HSD promotes the inflammatory response in zymosan A-induced peritonitis

**Figure 1:**
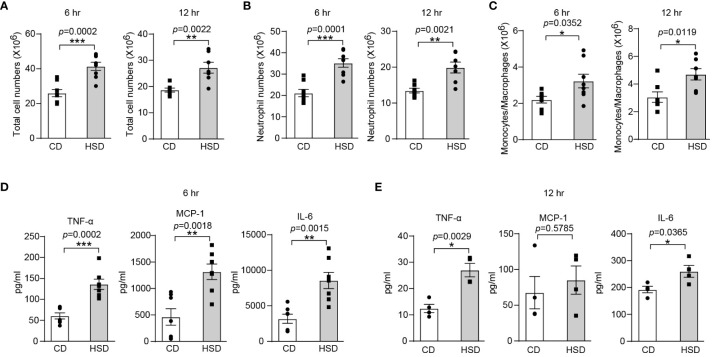
A HSD promotes the inflammatory response in zymosan A-induced peritonitis. After 8 weeks of treatment, zymosan A was administered (i.p.) at a dosage of 0.8 mg/mouse to induce peritonitis. **(A-C)**: The peritoneal fluid was collected 6 and 12 hr after injection. The total number of cells (**A**, 6 hr: n = 8, 12 hr: n = 7), neutrophils (**B**, 6 hr: n = 8, 12 hr: n=7) and monocytes/macrophages (**C**, 6 hr: n = 8, 12 hr: n=7) were determined. **(D, E)**: The level of inflammatory cytokines in the supernatant (**D**, 6 hr: n=7. E, 12 hr: n = 4) was determined by flow cytometry. The bars show the mean ± SEM, and the circles and squares represent individual mice. *p < 0.05, **p < 0.01, and ***p < 0.001 based on the unpaired two-tailed Student’s t test.

To investigate the proinflammatory effect of HSD, we used zymosan A to induce a classical acute inflammatory model of peritonitis After 8 weeks of CD or HSD, we supplemented the mice with zymosan A via intraperitoneal injection (i.p.) and then performed flow cytometry analysis and calculations to determine the impact of the HSD. A HSD significantly increased the number of total cells (6 hr: n = 8, *p* < 0.001; 12 hr: n = 7, *p* < 0.01), neutrophils (6 hr: n = 8, *p* < 0.001; 12 hr: n = 7, *p* < 0.01) and monocytes/macrophages (6 hr: n = 8, *p* < 0.05; 12 hr: n = 7, *p* < 0.05) in the peritoneum at both 6 and 12 hr after injection (**Figures 1A-C**). Furthermore, the expression of proinflammatory cytokines, such as TNF-α (6 hr: n = 7, *p* < 0.001; 12 hr: n = 4, *p* < 0.01), MCP-1 (6 hr: n = 7, *p* < 0.01; 12 hr: n = 4, *p* > 0.05), and IL-6 (6 hr: n = 7, *p* < 0.01; 12 hr: n = 4, *p* < 0.05), was also elevated following HSD treatment (**Figures 1D, E**). These findings strongly suggest that HSD not only promotes the infiltration of inflammatory cells but also increases the production of inflammatory cytokines in response to peritonitis. TNF-α and IL-6 are commonly used markers to assess the level of inflammation (26), and MCP-1 acts as a chemokine to attract circulating leukocytes to sites of inflammation (22). Taken together, these findings strongly suggest that a HSD exacerbates the inflammatory response in mice with zymosan A-induced peritonitis.

### The intestinal microbiota plays an important role in HSD-promoted inflammation

**Figure 2:**
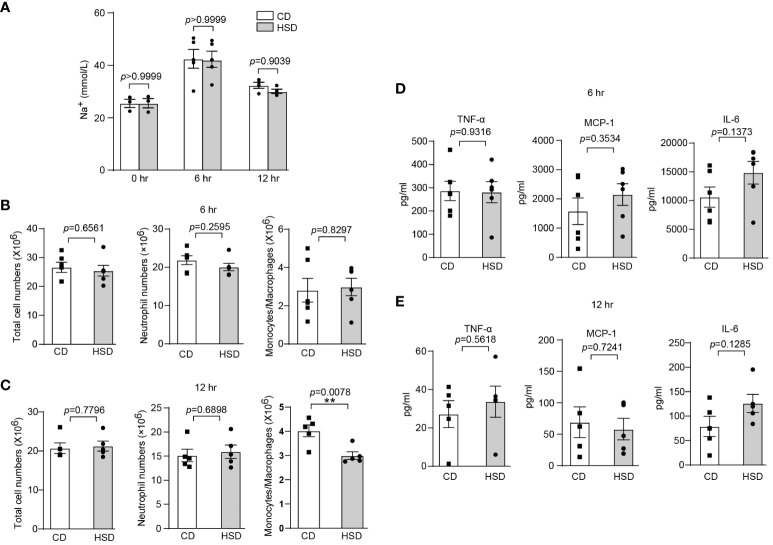
The intestinal microbiota plays an important role in HSD-promoted inflammation. **(A)**: After 8 weeks of treatment, the peritoneal fluid was collected before and after injection, and the concentration of Na^+^ in the supernatant was measured by biochemical analysis (n ≥ 3). **(B-E)**: Mice in both the HSD and CD groups were provided drinking water containing broad-spectrum antibiotics (ampicillin, 1 g/L; neomycin sulfate, 1 g/L; metronidazole, 1 g/L; and vancomycin, 500 mg/L) for 4 weeks ad libitum. After 8 weeks of treatment, zymosan A was administered, and the total number of cells, neutrophils numbers and monocytes/macrophages numbers **(B:** 6 hr, n = 6; **C**: 12 hr, n = 5), and the inflammatory cytokine level **(D:** 6 hr, n = 6, E: 12 hr, n = 5) in the supernatant were measured. The bars represent the mean ± SEM, and the circles and squares represent individual mice. Statistical analysis was performed via two-way ANOVA for multiple comparisons **(A)** or unpaired two-tailed Student’s t test **(B-E)**. *p < 0.05.

After confirming the proinflammatory effect of HSD in zymosan A-induced peritonitis, we aimed to further investigate the underlying mechanisms by which HSD exerts its effects. We first determined the concentration of Na+ in the peritoneal fluid supernatant at different time points. Interestingly, our biochemical analysis revealed no significant difference in the Na+ concentration (n ≥ 3, *p* > 0.05) between the HSD and CD groups (**Figure 2A**), indicating that the promotion of inflammation by HSD is not mediated by Na+ accumulation in the peritoneal cavity. Considering this result, we hypothesized that HSD might accelerate inflammation through indirect mechanisms, such as modulating the gut microbiota. To test this hypothesis, both the HSD and CD groups of mice were administered broad-spectrum antibiotics for 4 weeks to deplete the intestinal microbiota (27). Following antibiotic treatment, we assessed the impact of antibiotics on the response to peritonitis Surprisingly, although the levels of several proinflammatory cytokine such as TNF-α were increased in both the HSD and CD groups after antibiotic treatment compared to that in the nonantibiotic treatment, we found no significant differences between the HSD and CD groups in terms of total cell count (6 hr: n = 6, *p* > 0.05; 12 hr: n = 5, *p* > 0.05), neutrophil (6 hr: n = 6, *p* > 0.05; 12 hr: n = 5, *p* > 0.05) and monocyte/macrophage infiltration (6 hr: n = 6, *p* > 0.05) (**Figures 2B, C**), or the expression of key proinflammatory cytokines, namely, TNF-α, MCP-1, and IL-6 (6 hr: n = 6, *p* > 0.05; 12 hr: n = 5, *p* > 0.05), at 6 and 12 hours post zymosan A injection (**Figures 2D, E**). These results strongly suggest that HSD-promotion of inflammation may occur through aberration of the gut microbiota.

### HSD alters the composition of the gut microbiota

**Figure 3:**
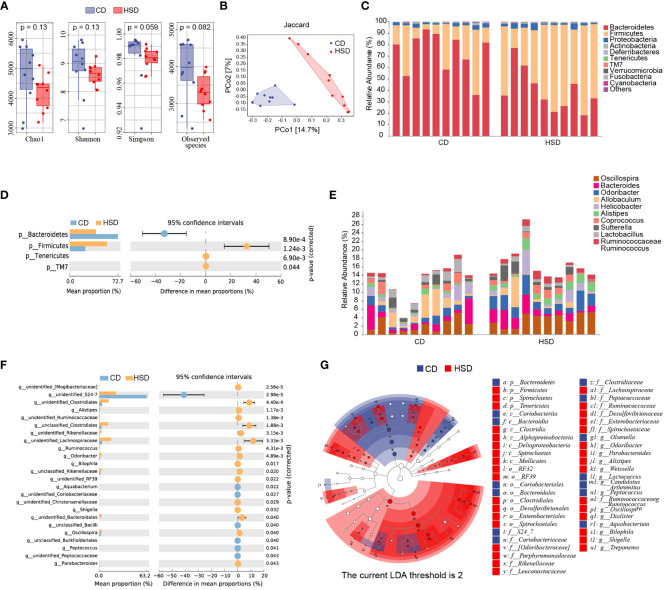
A HSD alters the gut microbiota composition. **(A)**: The Chao1, Shannon, Simpson and observed species indices of fecal microbiota alpha-diversity after 8 weeks of treatment were compared between the two groups. **(B)**: PCoA plots of the abundance Jaccard index of the fecal microbiota composition after 8 weeks of was compared between the two groups. **(C-F): **The fecal microbiota composition of HSD- and CD-treated mice at the phylum **(C)** and genus **(E)** levels was determined. Welch’s t test was used to compare bacterial abundance and diversity at the phylum **(D)** and genus levels **(F)**, respectively. **(G)**: LDA effect size (LEfSe, LDA>2) analysis of the fecal microbiota was performed. (n=10).

To gain a better understanding of the impact of HSD on the gut microbiota, we analyzed the diversity and composition of the fecal microbiota in mice by 16S rRNA. In the fecal samples of the two groups, 8-12 phyla, 34-48 families and 40-54 genera were detected. We observed a decrease in the richness and diversity of fecal bacteria after the mice were exposed to a HSD for 8 weeks, although this decrease was not statistically significant based on the α-diversity (**Figure 3A**). In addition, our analysis using Jaccard metrics (PERMANOVA of Jaccard distance: *pseudoF*=2.635541, *p*=0.001) showed a clear separation between the two groups, indicating significant modulation of the intestinal microbiota composition after HSD exposure (**Figure 3B**). Taxonomic composition analysis also revealed a significant shift in the composition of the gut bacteria. At the phylum level, the most predominant phyla were *Bacteroidetes* and *Firmicutes*. Furthermore, we found that *Firmicutes* was enriched in the group exposed to HSD, while *Bacteroidetes* was enriched in the CD group (**Figure 3C**). This difference in phylum composition was confirmed by conducting Welch’s t test, which revealed a statistically significant decrease in the relative abundance of *Bacteroidetes* and an increase in the relative abundance of *Firmicutes* after HSD consumption (**Figure 3D**; **Supplementary Table S3**). We also examined relative abundances of the gut bacteria at genus level. According to our analysis, the top 10 discriminatory taxa were *Oscillospira*, *Bacteroides*, *Odoribacter*, *Allobaculum*, *Helicobacter*, *Alistipes*, *Coprococcus*, *Sutterella*, *Lactobacillus*, and *Ruminococcaceae_Ruminococcus* (**Figure 3E**). Further investigation using Welch’s t test showed that genus_*Bacteroidales* family_*S24-7* was enriched in the CD group, whereas several taxa such as *Clostridiales*, *Alistipes*, *Lachnospiraceae*, *Odoribacter*, and *Oscillospira* were enriched in the HSD group.(**Figure 3F**; **Supplementary Table S3**). To identify the representative bacteria associated with HSD, we performed LEfSe analysis (LDA scores(log10)>2) and identified 47 bacteria from the phylum to genus levels. Consistent with our previous findings, we found that *Bacteroidetes* was enriched in the CD group, while *Firmicutes* was enriched in the HSD group. Additionally, we observed that several opportunistic pathogens, including *Parabacteroides*, *Alistipes*, *Dialister*, and *Bilophila*, had higher abundances in the HSD group, while *S24-7*, *Olsenella*, and *Candidatus_arthromitus* were significantly overrepresented in the CD group (**Figure 3G**; **Supplementary Figure S1**). Taken together, our results suggest that the richness and diversity of the intestinal microbiota decreased and that the composition of the gut bacteria was significantly altered after exposure to a HSD for 8 weeks.

### A HSD influences the metabolome of the gut microbiota

**Figure 4:**
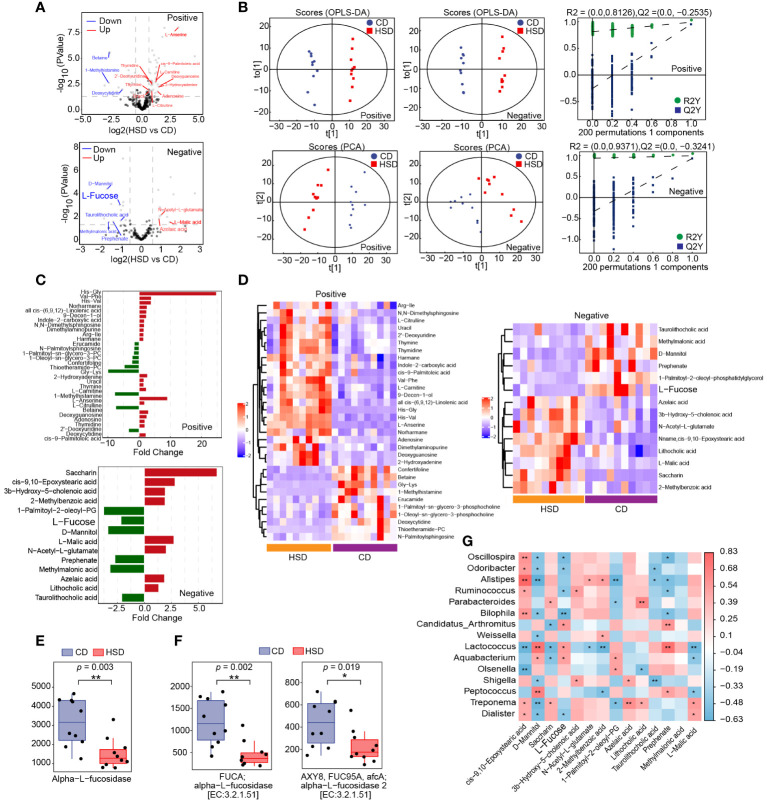
A HSD influences the metabolome of the gut microbiota. **(A, B)**: The volcano plot **(A)** and OPLS-DA **(B)** show the differences in metabolites between HSD and CD groups. **(C, D):** Significantly differentially abundant metabolites between the HSD and CD group were identified according to an OPLS-DA VIP > 1 and P value < 0.05 and are shown based on fold change **(C)** and HCA **(D)**. **(E, F)**: Based on metaCyc **(E)** and KEGG **(F)**, Phylogenetic Investigation of Communities by Reconstruction of Unobserved States (PICRUSt2) analysis was used to determine the abundance of fucosidase in the two groups respectively, and *p < 0.05 and **p <0.01 were calculated using the Kruskal−Wallis test for comparison. **(G)**: Spearman’s correlation analysis of microbes and metabolites was performed. (n=10) Red represents a positive correlation, and blue represents a negative correlation between microbes and metabolites. *P* value is the value of the correlation test, the lower the *p* value, the higher the accuracy of the verification of the verification results. *p < 0.05 and **p <0.01.

After 8 weeks of treatment, fecal samples were collected from mice in both the HSD group and the CD group for nontargeted LC-MS/MS-based metabolomics analysis. This analysis resulted in the identification of 284 metabolites in positive ion mode and 142 metabolites in negative ion mode. Volcano plots were constructed to graphically illustrate the differentially accumulated and significantly changed metabolites in the two groups based on fold change analysis in both ion modes (**Figure 4A**). Additionally, orthogonal partial least-squares discriminant analysis (OPLS-DA) revealed a clear separation, which indicated that there was a substantial difference between the HSD group and the CD group in terms of metabolite profiles (**Figure 4B**). These findings convincingly demonstrated that the HSD had a profound impact on the fecal metabolome. Moreover, utilizing the criteria of an OPLS-DA VIP > 1 and a P value < 0.05, we further narrowed our focus to 32 special metabolites identified in positive ion mode and 14 metabolites identified in negative ion mode (**Figures 4C, D**; **Supplementary Table S1-2**). Among these metabolites, excluding those that were undefined, we identified 14 metabolites in the positive ion mode; 11 metabolites had increased abundance, including 2-hydroxyadenine, uracil, thymine, L-carnitine, L-serine, L-citrulline, deoxyguanosine, adenosine, thymidine, 2’-deoxyuridine and cis-9-palmitoleic acid, and 3 metabolites had decreased abundance, including 1-methylhistamine, betaine and deoxycytidine, in the HSD group compared to those in the CD group. Similarly, in negative ion mode, 9 metabolites were selected, 4 of which were enriched in the HSD group; these included L-malic acid, N-acetyl-L-glutamate, azelaic acid and lithocholic acid. Five of these metabolites were enriched in the CD group, including L-fucose, D-mannitol, prephenate, methylmalonic acid and taurolithocholic acid.

### The gut microbiota metabolite L-fucose contributes to HSD-induced increases in inflammation

Subsequently, we aimed to identify intestinal metabolites that may contribute to HSD-promoted increase in inflammation. Excluding the unidentified metabolites, eight gut microbiota metabolites, namely 1-methylhistamine, betaine, D-mannitol, deoxycytidine, methylmalonic acid, prephenate, L-fucose and taurolithocholic acid, were found to be downregulated by HSD. Among them, only L-fucose (28) and betaine were reported to have anti-inflammatory activity (29). However, dietary betaine may also serve as a substrate for bacteria to form trimetlylamine and presumably trimetlylamine oxide which is associated with the increased risk of stroke, heart attack and death (30). Therefor we choose to further investigate the contributions of L-fucose to HSD-induced increases in inflammation.

L-fucose is typically found in glycans and can be released from these molecules by the gut microbiota via the enzyme fucosidase. Once liberated, L-fucose can be further metabolized and utilized by the body (31, 32) Therefore, we first investigated the impact of a HSD on the gene abundance of *fucosidase* in the gut microbiota by comparing the abundance of this gene sequences in the two groups using phylogenetic investigation of communities by reconstruction of unobserved states (PICRUSt2) analysis and found a significant decrease in the abundance of the fucosidase gene in the HSD group (metaCyc: Alpha-L-fucosidase, *p*<0.01; KEGG: alpha-L-fucosidase, *p*<0.01, FDR =0.004; alpha-L-fucosidase 2, *p*<0.05, FDR = 0.019) (**Figures 4E, F**). Additionally, we further investigated the relationship between the genus level flora identified by LEfSe and the metabolites identified in negative ion mode by Spearman’s correlation analysis (**Figure 4G**). The results showed that the level of L-fucose was significantly correlated with a more abundant flora, negatively correlated with the dominant flora in the HSD group, and positively correlated with the dominant flora in the CD group. This correlation suggested that these microbes are involved in the metabolism of L-fucose. Therefore, we hypothesized that the deficiency of fucosidase in the gut microbiota led to a decreased in the production of L-fucose in the HSD group.

**Figure 5:**
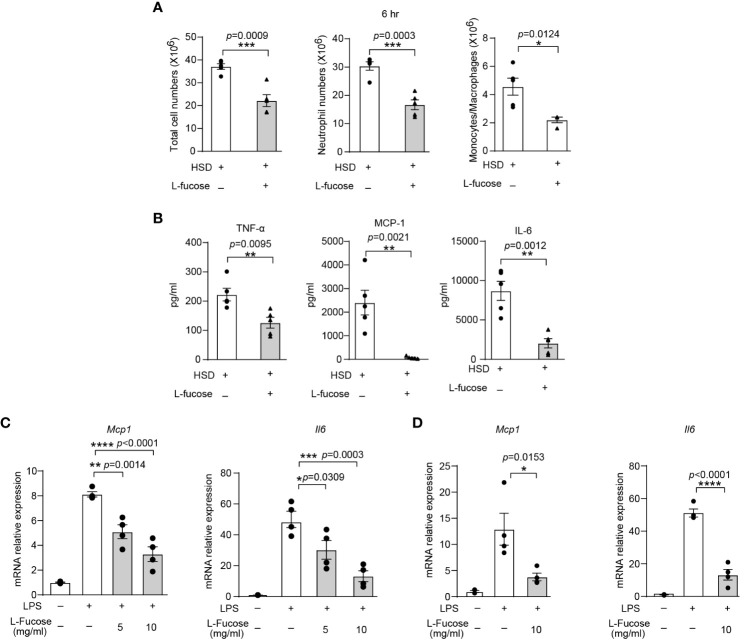
The gut microbiota metabolite L-fucose contributes to HSD-promoted inflammation. After 8 weeks of treatment, zymosan A was administered to induce peritonitis. **(A)**: The peritoneal fluid was collected 6 hr after injection. Total cell numbers, neutrophil numbers and monocytes/macrophages numbers were measured (n = 5). **(B)**: The level of inflammatory cytokines in the supernatant was determined by flow cytometry (n = 5). **(C, D):** RAW264.7 **(C)** and PMs **(D)** were stimulated with LPS and were treated with different concentrations of L-fucose for 4 hr *in vitro* (n = 6). The results are were expressed as mean ± SEM, and circles and triangles represent individual mice in **(A, B)**. Data are representative of at least two independent experiments; the circles represent technical replicates, and the results are expressed as the mean ± SEM in C and **(D)** Statistical analysis was performed via unpaired two-tailed Student’s t test **(A, B)** or one-way ANOVA for multiple comparisons **(C, D)**. *p < 0.05, **p < 0.01, ***p < 0.001, and ****p < 0.0001.

To verify the effectiveness of L-fucose in reducing inflammation *in vivo*, we conducted experiments using zymosan A-induced peritonitis in mice. The results showed that mice receiving both HSD and L-fucose simultaneously had significantly fewer total cells (n = 5, *p* < 0.001), neutrophils (n = 5, *p* < 0.001) and monocytes/macrophages (n = 5, *p* < 0.05) in the peritoneum (**Figure 5A**). Additionally, the expression levels of inflammatory markers such as TNF-α, MCP-1, and IL-6 (n = 5, *p* < 0.01) in the peritoneal fluid were reduced (**Figure 5B**). We further assessed the effects of L-fucose on RAW264.7 cells and peritoneal macrophages (PMs). The results of quantitative PCR demonstrated that the levels of *Mcp1* (5 mg/ml L-fucose: *p* < 0.01, 10 mg/ml L-fucose: *p* < 0.0001) and *Il6* (5 mg/ml L-fucose: *p* < 0.05, 10 mg/ml L-fucose: *p* < 0.001) were decreased in a concentration-dependent manner with L-fucose treatment in RAW264.7 cells (**Figure 5C**). Similarly, the relative expression of *Mcp1* (10 mg/ml L-fucose: *p* < 0.05) and *Il6* (10 mg/ml L-fucose: *p* < 0.0001) was also reduced in PMs (**Figure 5D**). These findings show that L-fucose can alleviate inflammation both *in vivo* and *in vitro*.

### Fucoidan, which is rich in L-fucose, alleviates HSD-promoted inflammation

**Figure 6:**
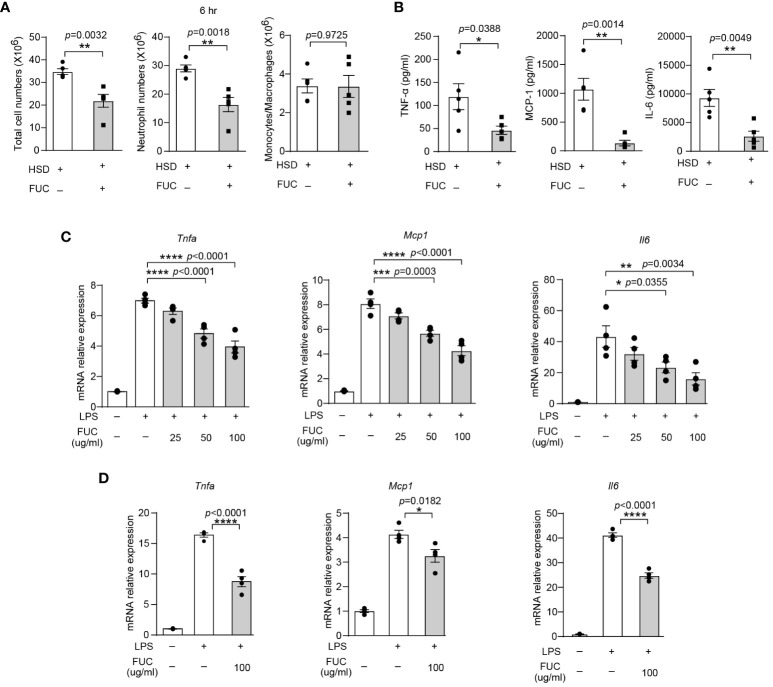
Fucoidan alleviates HSD-promoted inflammation. After 8 weeks of treatment, zymosan A was administered to induce peritonitis. **(A)**: The peritoneal fluid was collected 6 hr after peritoneal injection. The total cell number of cells, neutrophils numbers, and monocytes/macrophages numbers were measured (n = 5). **(B)**: The level of inflammatory cytokines in the supernatant was determined by flow cytometry (n = 5). **(C, D)**: RAW264.7 **(C)** and PM **(D)** cells were stimulated with LPS and treated with different concentrations of L-fucose for 4 hr *in vitro* (n=3). The results are expressed as the mean ± SEM, and circles and triangles represent individual mice in **(A, B)**. Data are representative of at least two independent experiments; the circles represent technical replicates, and the results were expressed as the mean ± SEM in **(C, D)**. Statistical analysis was performed via unpaired two-tailed Student’s t test **(A, B)** or one-way ANOVA for multiple comparisons **(C, D)**. * *p*<0.05, **p < 0.01, ***p < 0.001, and ****p < 0.0001.

Previous studies have reported that L-fucose is the primary component of fucoidan, a compound derived from seaweed (33). The abovementioned data revealed that L-fucose could alleviate inflammation caused by HSD. This finding led to curiosity about whether fucoidan could also possess anti-inflammatory properties similar to L-fucose. To investigate whether fucoidan could ameliorate the exacerbation of inflammation caused by HSD, we analyzed the cells and cytokines present in the peritoneal fluid of both the fucoidan-treated (FUC) group and the HSD group 6 hr after zymosan A injection by flow cytometry. The results revealed that compared with those in the HSD group, the number of total cells (n = 5, *p* < 0.01) and neutrophils (n = 5, *p* < 0.01) in the peritoneal fluid was lower in the fucoidan-treated group (**Figure 6A**). Moreover, the levels of the inflammatory cytokines TNF-α (n = 5, *p* < 0.05), MCP-1 (n = 5, *p* < 0.01), and IL-6 (n = 5, *p* < 0.01) in the supernatant of the FUC group were decreased compared to those in the HSD group (**Figure 6B**). Additionally, *in vitro* experiments using RAW264.7 cells and PMs were conducted, the results of which further supported these findings. The relative mRNA expression of *Tnfa* (for RAW264.7 cells: 50μg/ml, *p* < 0.0001; 100μg/ml, *p* < 0.0001; and PM, 100μg/ml, *p* < 0.0001); *Mcp1* (for RAW264.7 cells: 50μg/ml, *p* < 0.001; 100μg/ml, *p* < 0.0001; and PM, 100μg/ml, *p* < 0.05); and *Il6* (for RAW264.7 cells: 50μg/ml, *p* < 0.05; 100μg/ml, *p* < 0.01; and PM, 100μg/ml, *p* < 0.0001) were decreased (**Figures 6C, D**). Overall, these results suggest that, similar to its main component L-fucose, fucoidan, which has anti-inflammatory properties, can alleviate inflammation aggravated by HSD. .

## Discussion

HSDs have attracted much attention in recent years and are reportedly associated with many diseases, such as hypertension, cardiovascular disease, chronic kidney disease, and autoimmune diseases (10). Studies have suggested that inflammation plays a crucial role in the development of diseases induced by HSDs. Zymosan A has been found to activate immune cells known as macrophages and trigger the activation of NF-κB, thus leading to inflammation (34). To investigate the inflammatory effects of a HSD, we utilized a classic model of acute inflammation called peritonitis that was induced by injecting zymosan A into the peritoneal cavity. This model was characterized by the infiltration of inflammatory cells, particularly neutrophils, and an increase in the production of inflammatory molecules. The findings of this study demonstrated that, after being subjected to a HSD, there was a significant increase in the number of total cells and neutrophils in the peritoneal cavity, as well as the elevated levels of inflammatory cytokines TNF-α and IL-6. These results indicate that HSD consumption promotes inflammation in zymosan A-induced acute peritonitis. Furthermore, based on the analysis of Na+ concentration and treatment with an antibiotic cocktail, we discovered that the inflammatory effect caused by HSD was indirect rather than direct, and we hypothesized that the alteration of the intestinal microbiota could be the underlying mechanism of zymosan A-induced peritonitis.

The analysis of 16S rRNA revealed that the richness and diversity of fecal bacteria were decreased after the consumption of a HSD, which was consistent with a previous study (24). In addition, at the phylum level, the relative abundance of *Firmicutes* was significantly greater and that of *Bacteroidetes* was significantly lower in the HSD group than in the CD group, which was consistent with the microbiota composition in pathological states that have been reported in several studies (23, 35, 36). At the genus level, the relative abundance of *Bacteroidales* family *S24-7* was significantly decreased after the consumption of a HSD. Previous research by Qi C et al. has shown that *Bacteroidales* family *S24-7* plays a role in the release of extracellular DNA in the intestinal mucus layer of the small intestine. This process results in decreased proinflammatory activity by inducing low levels of TNF-α/IL-10, IL-6/IL-10, and IL-12, which help maintain immune homeostasis in the gut (37). Another study demonstrated that *S24-7* improved the symptoms of mice with dextran sulfate sodium (DSS)-induced colitis (38). Moreover, LEfSe analysis revealed that opportunistic pathogens such as *Parabacteroides, Alistipes, Dialister*, and *Bilophila* were enriched in the HSD group, while *Olsenella* and *Candidatus_Arthromitus* were significantly overrepresented in the CD group at the genus level. *Parabacteroides* is enriched in early hepatocellular carcinoma versus liver cirrhosis (39) and *Parabacteroides distasonis* promotes the symptoms of amyotrophic lateral sclerosis (40) The abundance of *Alistipes*. spp is positively related to hepatic inflammatory or oxidative stress markers (41) and is enriched in patients with type 2 diabetes or those fed a high-fat diet (42). The abundance of the genus *Dialister* was significantly higher in inflamed ileal and colonic samples from spondyloarthritis patients than in noninflamed healthy control samples (43). Moreover, the abundance of *Dialister* was significantly higher in cancer lesion samples from patients with oral squamous cell carcinoma than in those from controls (44), and was more abundant in gastric cancer than in superficial gastritis, atrophic gastritis, intestinal metaplasia (45). *Bilophila* has been shown to play a role in exacerbating the development of inflammatory bowel disease by reducing sulfite to hydrogen sulfide through the sulfite reductase enzyme (46). Additionally, *Olsenella* has been associated with alleviating metabolic syndromes or alleviating DSS-induced inflammation (47). *Candidatus_arthromitus*, a commensal bacterium, is essential for inducing the postnatal maturation of gut immune functions, including the innate and adaptive immune responses, in mice (48). In addition, this bacterium is absent in the ileum of mice fed a high-fat diet (49). Taken together, these findings demonstrate that HSD consumption disrupts the balance of the gut microbiota and promotes disease development. Recent reports showed that gut microbiota dysregulation could be associated with low-grade inflammation and lots of pathological conditions by metabolites indirectly (50, 51). Therefore, we analyzed the alteration of the metabolites in the stools of the mice.

Nontargeted LC-MS/MS-based metabolomics analysis revealed that L-fucose levels were significantly lower in the HSD group than in the CD group, and this reduction in L-fucose was closely linked to changes in the composition of the gut bacteria caused by HSD. Furthermore, the potential impact of L-fucose on inflammation showed that the inflammatory response was significantly reduced in the group of mice that received L-fucose treatment. These findings suggested that L-fucose plays a critical role in modulating inflammation in the context of HSD consumption. Additionally, we observed a decrease in the levels of fucosidase, an enzyme responsible for releasing L-fucose from glycan, in the HSD group. These findings suggested that a HSD might hinder the production of L-fucose by reducing the abundance of bacteria that possess fucosidase activity. This reduction in L-fucose production could promote inflammation. Previous studies have reported that L-fucose has anti-inflammatory properties. For example, L-fucose inhibits macrophage M1 polarization, inactivates the NLRP3 inflammasome, and decreases the levels of TNF-α, IL-1β, and IL-6 in mice with DSS-induced colitis (28). L-fucose also blocks MIF/MAF-induced priming of alveolar macrophages for an oxidative burst (52). Moreover, it is demonstrated that L-fucose can be detected in the bloodstream after oral treatment in rats (53). Consistent with these previous reports, in the present study, we revealed that L-fucose treatment significantly reduced the release of IL-6 and MCP-1 in mice with HSD treatment in RAW264.7 cells and PMs from HSD-treated mice. L-fucose can be a nutrient for the colonization of microbes, including Bifidobacteria, and can change the composition of the microbiota; moreover, in the presence of gut microbes, L-fucose can alleviate DSS-induced colitis by regulating bile acid metabolism (32, 54, 55). In addition, L-fucose can be utilized by various bifidobacterial species to form 1,2-propanediol, which is a precursor for intestinal propionate formation (56). The production of propionic acid may ameliorate intestinal inflammation by regulating Reg3-associated epithelial homeostasis (57) or increasing the number of Treg cells (58). Overall, these findings suggest that L-fucose could be a potential therapeutic agent for reducing inflammation in the context of a HSD. However, further research is needed to elucidate the precise mechanisms by which L-fucose exerts its anti-inflammatory effects and to explore its potential for clinical application.

Fucoidan, a sulfated polysaccharide, is a compound that can be extracted from brown algae and sea cucumbers (59, 60). Fucoidan is mainly composed of fucose and sulfate, with smaller amounts of galactose, xylose, mannose, and uronic acids (61) Previous studies have indicated that fucoidan, which contains relatively high levels of fucose and sulfates, tends to have more significant bioactive effects than other products (62). In our research, we conducted experiments that demonstrated the potential of fucoidan in alleviating inflammation caused by HSD in mice. We also observed that fucoidan reduced acute inflammatory responses in macrophages. Interestingly, we discovered that the effective dose of fucoidan is lower than that of L-fucosee, which might be related to another main component of fucoidan, sulfate. A study of three fucoidans from Ecklonia cava suggested that the sulfate and fucose contents of fucoidan might contribute to the inhibition of NO production by macrophages (33). However, much work still needs to be done to fully understand the mechanisms through which HSD consumption promotes inflammation. For instance, we did not assess the concentration of L-fucose in the blood or peritoneal fluid after the mice consumed a HSD or received L-fucose or fucoidan treatment. The composition of fucoidan, excluding fucose, has also not been thoroughly investigated. These aspects should be further explored in future studies.

## Conclusion

**Figure 7:**
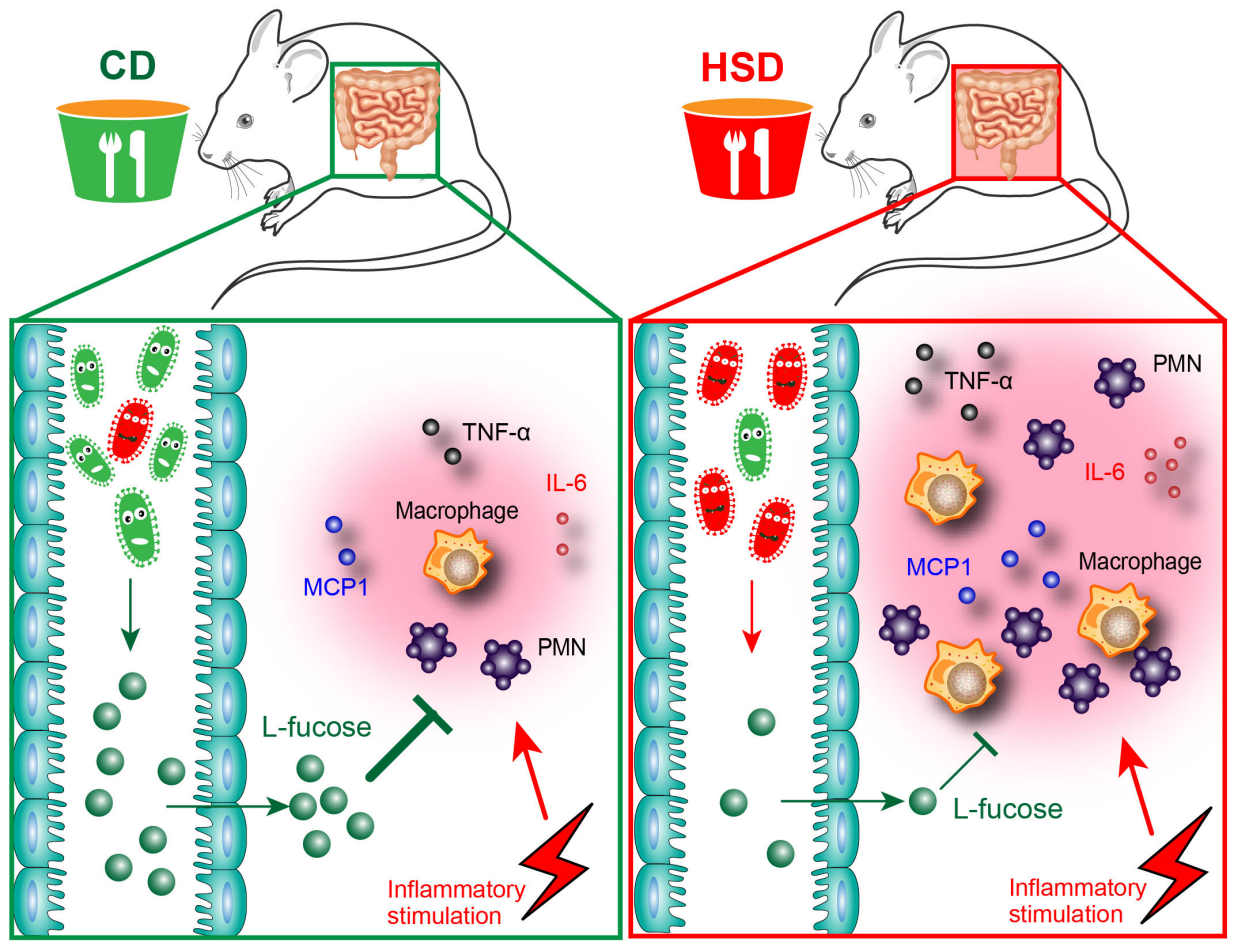
Hypothesis schema depicting the mechanism of L-fucose in HSD-promoted inflammation. Mice received CD or HSD for 8 consecutive weeks. The CD-fed mice possess a normal gut microbiota, which leads to a higher abundance of an intestinal microbial metabolite L-fucose. L-fucose has anti-inflammatory properties, and when CD-fed mice are stimulated with inflammatory injuries, the inflammatory response in CD-fed mice is controlled at a reasonable level. On the contrary, the HSD consumption altered the composition of gut microbiota in the HSD-fed mice and significantly reduced the abundance of L-fucose. When HSD-fed mice are stimulated with inflammatory injuries, reduced L-fucose levels in the gut contributed to a more substantial inflammatory response, as evidenced by the increased accumulation of inflammatory cells and elevated levels of inflammatory cytokines including TNF-α, IL-6, and MCP-1.

Overall, in our study, we demonstrated that HSD consumption decreased the diversity and richness of the intestinal microbiota and disrupted the composition of the intestinal microbiota and metabolites which play important roles in the promotion of peritonitis (**Figure 7**). Both L-fucose and fucoidan can alleviate the proinflammatory effect of HSD in zymosan A- induced peritonitis. The findings of this study shed new light on the underlying mechanisms behind the proinflammatory effects of HSD. Furthermore, the identification of potential drug candidates, such as L-fucose and fucoidan, opens up new avenues for clinical therapy.

## Limitations

Although in the present study we revealed that HSD-reduced the abundance of the microbial metabolite L-fucose and contributes to HSD-increases in inflammation, the underlying mechanisms remain unclear; in particular, how HSD decreases gut L-fucose levels and how L-fucose alleviates inflammation remain unknown. Moreover, the translational application of L-fucose and fucoidan for HSD-related disorders warrants further investigation.

## Methods

### Mice

Male C57BL/6J mice (6-8 weeks old, 22-26 g) were purchased from SPF (Beijing) BIOTECHNOLOGY Co., Ltd (Beijing, China) and the permission number was SCXK (jing) 2019-0010. All mice were maintained under 12-hr light/dark cycles and standard conditions for temperature and humidity in specific pathogen-free conditions at the central animal facility at the Third Military Medical University.

### HSD and acute zymosan A induced peritonitis induction

After 1 week of acclimatization, the mice received normal chow and sterile tap water ad libitum (CD) or sodium-rich chow containing 4% NaCl and sterile tap water containing 1% NaCl ad libitum (HSD) for 8 consecutive weeks as previously described (14). After 8 weeks, the mice were treated with zymosan A (0.8 mg/mouse) by intraperitoneal injection to induce acute peritonitis.

### Collection and analysis of the sodium concentration

Mice were fed a CD or HSD for 8 weeks and then sacrificed (0 hr) or treated with zymosan A (0.8 mg/mouse) by intraperitoneal injection (6 hr or 12 hr). A total of 1 ml of 5% glucose was injected into the peritoneal cavity of the mice, the abdomen was massaged, and the suspension of peritoneal fluid was collected for biochemical analysis at Xinqiao Hospital of Army Medical University.

### Cell calculation

The cells were filtered through 200 mesh nylon and then treated with trypan blue for 3 min. After trypan blue treatment, 10 μl of cell suspension was collected for analysis via biological microscopy.

### Antibiotic treatment

At the beginning of the 5th week of CD or HSD supplementation, the mice were provided with drinking water containing an antibiotic cocktail (ampicillin, 1 g/L; neomycin sulfate, 1 g/L; metronidazole, 1 g/L; and vancomycin, 500 mg/L; Sangon Biotech (Shanghai) Co., Ltd.) for 4 weeks ad libitum in the meantime (27). The antibiotics were renewed every other day. After 4 weeks of antibiotic treatment, the mice were treated with zymosan A, and the peritoneal fluid was collected for further experiments.

### Flow cytometry

The suspension and cells in the peritoneal fluid were both analyzed via flow cytometry. All the fluorescence-labeled antibodies used in the flow cytometry assays in this study were purchased from Biolegend, including an anti-mouse CD16/32 antibody (101302 [clone 93]; BioLegend), an APC anti-mouse/human CD11b antibody (101212 [clone M1/70]; BioLegend),a FITC-conjugated anti-mouse Ly6G antibody (M100L8-02E[clone 1A8]; Sungene Biotech) for detection of CD11b^+^Ly6G^+^ neutrophils and CD11b^+^Ly6G^-^ monocytes/macrophages. A Biolegend LEGENDplexTM Multi-Analyte Flow Assay Kit (Mouse TNF-α Capture Bead A7, 13× 740153; Mouse MCP-1 Capture Bead A8, 13×, 740155; and Mouse IL-6 Capture Bead B4, 13× 740159; BioLegend) was used for detection of cytokines. For staining specific cells or cytokines, all the analyses were carried out according to the manufacturer’s instructions. Cell and cytokine data were acquired on a FACSCantoTM ǁ flow cytometer (BD Biosciences) and BD Accuri C6 Plus, and analyzed separately with FlowJo and LEGENDplex v8.0 software, separately.

### DNA extraction and 16S rRNA sequencing

Total genomic DNA was extracted from the samples using an OMEGA Soil DNA Kit (M5635-02) (Omega Bio-Tek, Norcross, GA, USA), and measured using a NanoDrop NC2000 spectrophotometer (Thermo Fisher Scientific, Waltham, MA, USA) and agarose gel electrophoresis. PCR amplification of the V3–V4 region of the bacterial 16S rRNA gene was performed using the forward primer 338F (5’-ACT CCT ACG GGA GGC AGC A-3’) and the reverse primer 806R (5’-GGA CTA CHV GGG TWT CTA AT-3’). Sample-specific 7-bp barcodes were incorporated into the primers for multiplex sequencing. Thermal cycling consisted of initial denaturation at 98°C for 5 min, 25 cycles of denaturation at 98°C for 30 s, annealing at 53°C for 30 s, and extension at 72°C for 45 s; and a final extension of 5 min at 72°C. PCR amplicons were purified with Vazyme VAHTSTM DNA Clean Beads (Vazyme, Nanjing, China) and quantified using the Quant-iT PicoGreen dsDNA Assay Kit (Invitrogen, Carlsbad, CA, USA). After the individual quantification step, amplicons were pooled in equal amounts, and paired 2x250 bp sequencing was performed using the Illumina MiSeq platform with MiSeq Reagent Kit v3 at Shanghai Personal Biotechnology Co., Ltd (Shanghai, China).

### Sequencing data analysis

Microbiome bioinformatics was performed with QIIME2 2019.4 according to official tutorials with slight modifications (https://docs.qiime2.org/2019.4/tutorials/) (63). Briefly, raw sequence data were demultiplexed using the demux plugin followed by primer cutting with the cutadapt plugin. The sequences were then quality filtered, denoised, and merged and chimera were removed using the DADA2 plugin. Nonsingleton amplicon sequence variants (ASVs) were aligned with MAFFT and used to construct a phylogenetic tree with fasttree2. Alpha-diversity metrics, beta-diversity, and different statistical analyses were performed using QIIME2 (2019.4). The relationships between the HSD and microbiota phylogeny were investigated by principal coordinate analysis (PCoA) of the Jaccard distances, and the significance of differences of microbiota structure among groups was assessed by permutational multivariate analysis of variance (PERMANOVA) using QIIME2, and the number of permutations run with PERMANOVA is 999. The taxonomy composition and abundances were visualized using MEGAN and Welch’s t test was used to compare bacterial abundance at the phylum and genus levels. Linear discriminant analysis effect size (LEfSe) was performed to identify differentially abundant taxa across groups using the default parameters. Microbial functions were predicted by PICRUSt2 (Gavin M. Douglas, et al., preprint) via the MetaCyc (https://metacyc.org/) and KEGG (https://www.kegg.jp/) databases. The difference between the CD group and HSD groups given alpha-l-fucosidase was assessed by the Kruskal−Wallis test and visualized using a boxplot on a free online platform called genescloud (https://www.genescloud.cn).

### Fecal sample collection and preparation

Fecal samples were collected immediately after the mice had experienced natural bowel movements and were quickly frozen in liquid nitrogen. Then, the fecal samples were thawed at 4°C and mixed with cold methanol/acetonitrile/water (2:2:1,v/v). The mixture was vortexed, cryo-sonicated for 30 min, frozen at -20°C for 10 min and centrifuged for 20 min (14000 g, 4°C). The supernatant was dried in a vacuum centrifuge. For LC−MS analysis, the samples were redissolved in 100 μL of acetonitrile/water (1:1, v/v) solvent, vortexed, and centrifuged for 15 min (14000 g, 4°C). The supernatant was collected for further investigation.

### LC−MS/MS analysis

Analyses were performed using a UHPLC (1290 Infinity LC, Agilent Technologies) coupled to a quadrupole time-of-flight (AB Sciex TripleTOF 6600) at Shanghai Applied Protein Technology Co., Ltd. For HILIC separation, samples were analyzed using a 2.1 mm × 100 mm ACQUITY UPLC BEH Amide 1.7 µm column (Waters, Ireland). In both the ESI-positive and ESI-negative modes, the mobile phase contained A = 25 mM ammonium acetate and 25 mM ammonium hydroxide in water, and B=acetonitrile. The gradient was 95% B for 0.5 min and was linearly reduced to 65% in 6.5 min; then, B was reduced to 40% in 1 min, kept at that level for 1 min, and then increased to 95% in 0.1 min. The whole analysis process was performed at 4°C. QC samples were used to evaluate the stability of the system and the reliability of the experimental data.

An AB Triple TOF 6600 mass spectrometer was used for primary and secondary spectra acquisition. The ESI source conditions were set as follows: Ion Source Gas1 (Gas1) 60, Ion Source Gas2 (Gas2) 60, curtain gas (CUR) 30, source temperature 600°C, and IonSapary Voltage Floating (ISVF) ± 5500 V. In MS-only acquisition, the instrument was set to acquire signals over the m/z range of 60-1000 Da, and the accumulation time for the TOF-MS scan was set at 0.20 s/spectra. In auto MS/MS acquisition, the instrument was set to acquire masses in the m/z range of 25-1000 Da, and the accumulation time for the product ion scan was 0.05 s/spectra. The product ion scan was acquired using information-dependent acquisition (IDA) with high-sensitivity mode selected. The parameters were set as follows: the collision energy (CE) was fixed at 35 V with ± 15 eV; the declustering potential (DP) was 60 V (+) and −60 V (−); isotopes within 4 Da were excluded; and 10 candidate ions were monitored per cycle.

### Metabolomics data analysis and statistical analyses

The raw MS data were converted to mzXML files using ProteoWizard MSConvert before importing them into freely available XCMS software. Fold change analysis (FC > 1.5 or FC < 0.67, *p* value < 0.05) was performed by using a volcano plot. The processed data, which were normalized to the total peak intensity, were analyzed by the R package (ropls) and subjected to orthogonal partial least-squares discriminant analysis (OPLS-DA). Sevenfold cross-validation and response permutation testing were used to evaluate the robustness of the model. The variable importance for the projection (VIP, VIP > 1) value of each variable in the OPLS-DA and the Student’s t test at the univariate level (*p* < 0.05) of each metabolite were used to investigate the significantly different metabolites between groups in the study. The results are displayed by a histogram. Hierarchical clustering analysis (HCA) was used to cluster the metabolites with similar functions together in the positive and negative ion modes. The relationships between important metabolites in negative ion mode and differentially abundant taxa at the genus level that were identified via LEfSe were analyzed by Spearman’s algorithm.

### Administration of L-fucose and fucoidan

Mice were randomly assigned to two different experimental groups: the HSD group and the L-fucose group. Both groups received sodium-rich chow containing 4% NaCl and sterile tap water supplemented with 1% NaCl ad libitum (HSD) for 8 weeks; the L-fucose group received 0.1% L-fucose in the water simultaneously (64).

Mice were randomly assigned to the HSD group or the fucoidan group, and all received sodium-rich chow containing 4% NaCl and sterile tap water containing 1% NaCl ad libitum (HSD) for 8 weeks. Mice in the fucoidan group were administered with fucoidan (100 mg/kg/day, 200 μl/mouse) once a day by gavage at the beginning of the third week while mice in the HSD group were administered with sterile tap water.

### The collection of PM

Wild-type C57BL/6J mice were intraperitoneally injected with 1 ml of 4% thioglycolic acid. After 72 hrs, the mice were sacrificed, and peritoneal fluid was collected.

### Cell incubation

RAW264.7 macrophages were seeded into twelve-well plates and divided into 4 groups. RAW264.7 macrophages were treated with or without 2 mg/ml LPS or with or without L-fucose (5 mg/ml, 10 mg/ml) for 4 hr. PMs from wild-type C57BL/6J mice were collected, seeded into twelve-well plates and divided into 3 groups. PMs were treated with or without 1 mg/ml LPS and with or without L-fucose (10 mg/ml) for 4 hr. The cells were collected to quantify the levels of IL-6, and MCP-1 by qPCR.

RAW264.7 macrophages were seeded into twelve-well plates and divided into 5 groups. RAW264.7 macrophages were treated with or without 2 mg/ml LPS or with or without fucoidan (25 mg/ml, 50 mg/ml, 100 mg/ml) for 4 hr. PMs were seeded into twelve-well plates and divided into 3 groups. The cells were treated with or without 1 mg/ml LPS and with or without fucoidan (100 mg/ml) for 4 hr. The cells were collected to quantify the levels of TNF-α, MCP-1, and IL-6 by qPCR respectively

### qPCR

Total RNA was extracted from treated and nontreated RAW264.7 cells or PMs with an RNA extraction kit (Flyjet Bio) according to the manufacturer’s instructions. Complementary DNA was synthesized using an equivalent amount of total RNA (1 μg) in a 10 μl reverse transcriptase reaction mixture using a cDNA synthesis kit (TaKaRa Biomedical Technology, Japan). Gene expression was analyzed by using SYBR Green (MedchemExpress). Gene expression for each sample was normalized to that of β-actin (*Actb*), and the differences were determined using the 2 ^ - (ΔΔCt) methods. The primer pairs used in this study were listed below: *Il6*: forward, 5’-AGT CCT TCC TAC CCC AAT TTC C-3’, reverse, 5’-TAA CGC ACT AGG TTT GCC GA-3; *Mcp1*: forward, 5’-AGC CAA CTC TCA CTG AAG CC-3’, reverse, 5’-TCT CCA GCC TAC TCA TTG GGA -3’; *Tnfa*: forward, 5’-GTC CCC AAA GGG ATG AGA AGT-3’, reverse, 5’-TTT GCT ACG ACG TGG GCT AC-3’; *Actb*: forward, 5’-GGC TGT ATT CCC CTC CAT CG-3’, reverse, 5’-CCA GTT GGT AAC AAT GCC ATG T-3’

### Statistics and reproducibility

Data are expressed as mean ± SEM unless otherwise indicated. Statistical comparisons were made in the GraphPad Prism 9.0 software using one-way or two-way ANOVA with Tukey’s post-hoc test for multiple comparisons (for groups across variables, with multiple comparisons between groups) or Student’s t-test for two groups meeting the normal distribution criteria. Data were subjected to Grubbs’ test to identify the presence of outlier data points. For all statistical analyses, the statistical significance was represented by a single asterisk (*p* < 0. 05), two asterisks (*p* < 0. 01), three asterisks (*p* < 0. 001), or four asterisks (*p* < 0. 0001). All experiments were repeated in at least duplicate, unless otherwise indicated. No statistical methods were used to predetermine sample size.

## Data availability statement

The datasets presented in this study can be found in online repositories. The names of the repository/repositories and accession number(s) can be found below: SRP461641 (SRA) and MTBLS9134 (Metabolights).

## Ethics statement

The animal study was approved by Laboratory Animal Welfare and Ethics Committee of the Third Military Medical University. The study was conducted in accordance with the local legislation and institutional requirements.

## Author contributions

WL: Data curation, Formal analysis, Methodology, Validation, Writing – original draft. PW: Data curation, Formal analysis, Methodology, Validation, Writing – original draft. TJ: Data curation, Formal analysis, Validation, Writing – original draft. JJ: Data curation, Formal analysis, Validation, Writing – original draft. BC: Data curation, Investigation, Methodology, Writing – original draft. TL: Data curation, Formal analysis, Validation, Writing – original draft. YL: Data curation, Formal analysis, Validation, Writing – original draft. JM: Data curation, Formal analysis, Validation, Writing – original draft. BL: Conceptualization, Funding acquisition, Supervision, Writing – review & editing. ZZ: Conceptualization, Funding acquisition, Methodology, Project administration, Supervision, Writing – review & editing.
