# Supplementary figures and images for: L-fucose and fucoidan alleviate high-salt diet-promoted acute inflammation
Source: Front Immunol. 2024 Mar 26;15:1333848. doi: 10.3389/fimmu.2024.1333848 (PMC11002173; doi:10.3389/fimmu.2024.1333848)

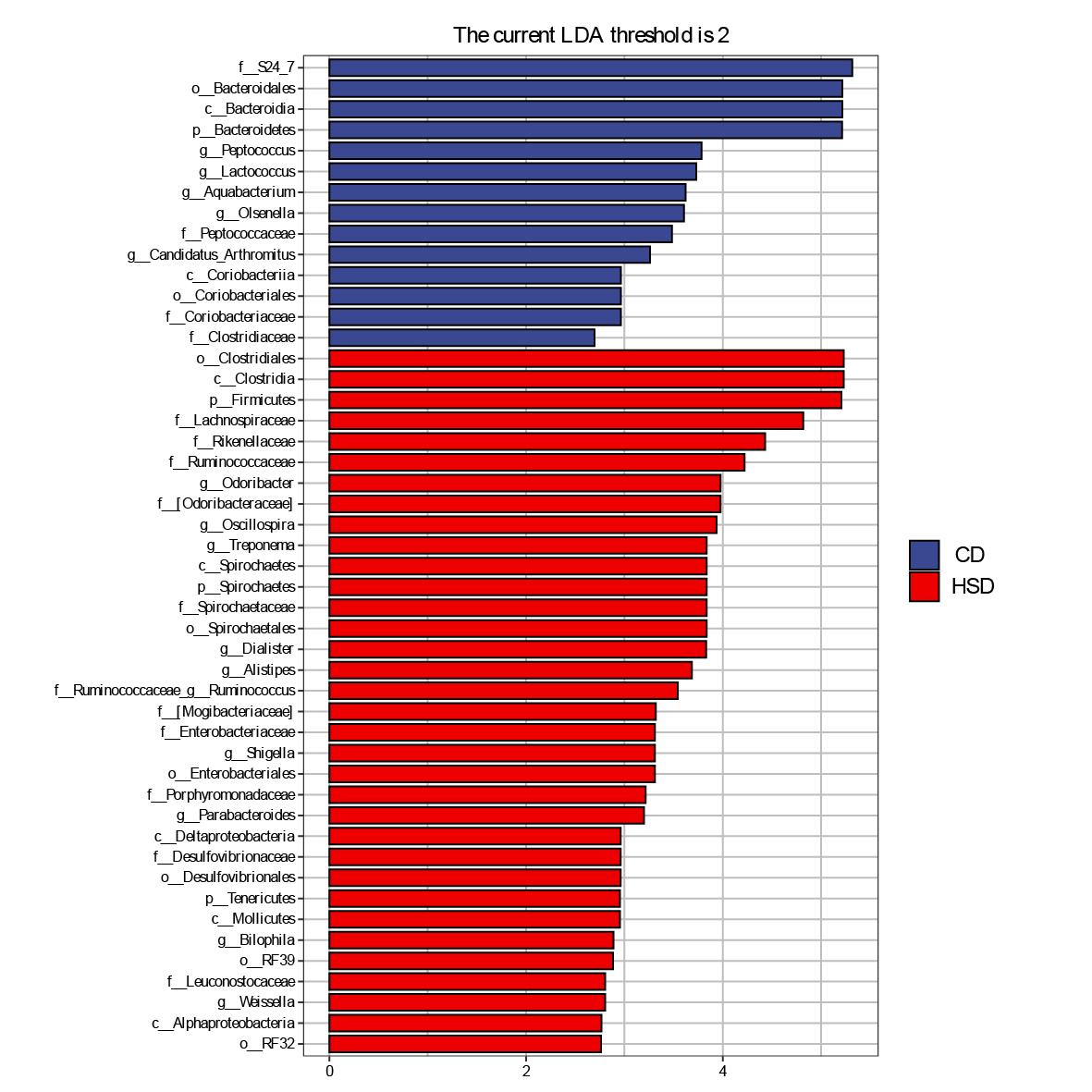

Supplement: Supplementary file 1 [file Image_1.jpeg]
